# Resveratrol Attenuates Oxalate-Induced Renal Oxidative Injury and Calcium Oxalate Crystal Deposition by Regulating TFEB-Induced Autophagy Pathway

**DOI:** 10.3389/fcell.2021.638759

**Published:** 2021-02-25

**Authors:** Yue Wu, Yang Xun, Jiaqiao Zhang, Henglong Hu, Baolong Qin, Tao Wang, Shaogang Wang, Cong Li, Yuchao Lu

**Affiliations:** ^1^Department of Urology, Tongji Hospital, Tongji Medical College, Huazhong University of Science and Technology, Wuhan, China; ^2^Institute of Urology, Tongji Hospital, Tongji Medical College, Huazhong University of Science and Technology, Wuhan, China

**Keywords:** nephrolithiasis, oxalate, resveratrol, autophagy, transcription factor EB

## Abstract

The oxidative injury of renal tubular epithelial cells caused by inflammation and oxidative stress induced by hyperoxaluria is an important factor in the kidney calcium oxalate (CaOx) stone formation. Resveratrol (RSV) has been reported to reduce oxidative injury to renal tubular epithelial cells, and autophagy is critical for the protective effect of resveratrol. However, the protective mechanism of RSV in oxalate-induced oxidative injury of renal tubular cells and the role of autophagy in this process are still unclear. In our study, glyoxylic acid monohydrate-induced rats were treated with or without resveratrol, and it was detected that the overexpression of oxidant species, CaOx crystal deposition, apoptosis level, inflammatory cytokines and osteoblastic-associated protein expression were reversed by resveratrol. Additionally, Resveratrol pretreatment significantly reversed oxalate -induced decline in cell viability, cell damage, oxidant species overexpression, and osteogenic transformation in normal rat kidney epithelial-like (NRK-52E) cells. Furthermore, we found that RSV pretreatment promoted intracellular LC3II upregulation, p62 downregulation, and autophagosome formation, whereas 3-methyladenine treatment reduced this effect. Moreover, RSV induced the expression of transcription factor EB (TFEB) in the nucleus of NRK-52E cells in a concentration-dependent manner. After transfection of NRK-52E cells with TFEB siRNA, we showed that the RSV-induced increase in TFEB expression and autophagosome formation were inhibited. Simultaneously, RSV-induced NRK-52E cells protection was partially reversed. These results suggested that RSV regulates oxalate-induced renal inflammation, oxidative injury, and CaOx crystal deposition *in vitro* and *in vivo* through the activation of a TFEB-induced autophagy.

## Graphical Abstract

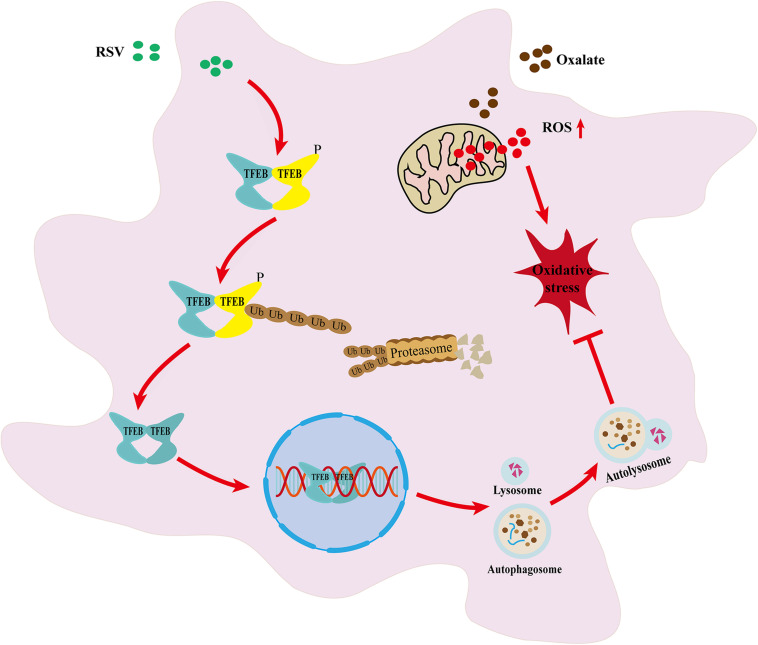

## Introduction

Kidney stone disease is a recurrent and lifelong disease that causes significant health and financial burden. The worldwide prevalence of kidney stone ranges from 7 to 13% in North America, from 5 to 9% in Europe, and from 1 to 5% in Asia (Sorokin et al., 2017). Worldwide, approximately 80% of kidney stones are calcium-containing stones (Kusmartsev et al., 2016). Although numerous studies have attempted to elucidate the mechanism of kidney stone formation, the exact details remain unclear. Hyperoxaluria is a known key factor in kidney stone formation (Coe et al., 2005). Injury induced by high concentrations of oxalate in renal tubular epithelial cells is related to oxidative stress and inflammation (Tsuji et al., 2016); these conditions not only contribute to the crystallization of calcium oxalate (CaOx) by providing material basis for its ectopic nucleation, but also facilitate the adhesion of CaOx crystals to renal tubular epithelial cells (Aggarwal et al., 2013; Khan, 2013). Thus, the inhibition of renal inflammation and oxidative stress may be a potential strategy to inhibit kidney stone formation and reduce stone recurrence (Davalos et al., 2010).

Resveratrol (RSV, trans-3,5,4′-trihydroxystilbene), a phenolic compound in the stilbene family, has strong antioxidant activity and is found in various plants (Frémont, 2000). Numerous studies have shown that resveratrol has protective and therapeutic effects against a variety of diseases, including diabetes, metabolic syndrome, cancer, and neurological and cardiovascular diseases (Cichewicz and Kouzi, 2002; Hou et al., 2019; Singh et al., 2019; Vervandier-Fasseur and Latruffe, 2019). Hong et al. (2013) previously found that RSV had biological antioxidant activity in the kidney and potential preventive effects against kidney stone formation, and Oksay et al. (2017) also found that RSV exerts protective effects in a rat model of hyperoxaluria. However, the method and exact mechanism of action remain unknown. Autophagy is a highly conserved intracellular self-digestion process that maintains cellular homeostasis by degrading aging or damaged proteins and organelles in lysosomes and plays an important role in a variety of diseases, including kidney disease (Dikic et al., 2010; Kim et al., 2013; Takabatake et al., 2014). Dysfunction in autophagy can lead to impaired mitochondrial function, the accumulation of reactive oxygen species (ROS), and oxidative stress in cells (Kimura et al., 2013). In addition, autophagy is critical to the RSV-induced protection, although the exact mechanism of RSV-induced autophagy and the related transcriptional regulatory mechanisms are still unclear (Li et al., 2014; Wang et al., 2014). However, recent studies have shown that the transcription factor EB (TFEB) is a major regulator of the autophagy-lysosome pathway and regulates the transcription and metabolism of organisms (Settembre et al., 2011, 2013). Additionally, a study showed that LC3 lipidation was critical for TFEB activation in a mouse model of lysosomal-damaged oxalate nephropathy. TFEB knockout in the proximal tubules of mice led to the progression of renal injury induced by oxalate crystals (Nakamura et al., 2020). Another study showed that the upregulation of mTOR (mechanistic target of rapamycin kinase) induced the inhibition of the upstream autophagy regulator TFEB, leading to autophagy deficiency and thus exacerbating the development of kidney stones (Unno et al., 2020).

Therefore, we hypothesized that autophagy was involved in the RSV-mediated antioxidant effects in renal tubular epithelial cells, and that RSV exerted protective effects against oxalate-induced oxidative injury in renal tubular epithelial cells and preventive effects against kidney stone formation through TFEB-mediated autophagy.

## Materials and Methods

### Reagents and Antibodies

The Cell Counting Kit 8 (CCK-8) (CK04) was purchased from Dojindo Molecular Technologies, Inc. (Kumamoto, Japan), the Annexin V-FITC Apoptosis Detection Kit (KGA108-1) was obtained from KeyGen Biotech (Jiangsu, China), and the ROS assay kit (S0033), lipid peroxidation malondialdehyde (MDA) assay kit (S0131), radioimmunoprecipitation assay (RIPA) lysis buffer (P0013B), phenylmethanesulfonyl fluoride (ST506), and bicinchoninic acid protein assay kit (P0012) were purchased from Beyotime Institute of Biotechnology (Jiangsu, China). RSV (R5010), methyl cellulose (MC) (M0512), and dihydroethidium (DHE) (D7008) were purchased from Sigma-Aldrich (St. Louis, MO, United States). Antibodies to osteopontin (OPN) (ab8448), bone morphogenetic protein 2 (BMP2) (ab14933), and interleukin 6 (IL-6) (ab9324), an oxalate assay kit (Colorimetric) (ab196990), and a calcium assay kit (Colorimetric) (ab102505) were purchased from Abcam (Cambridge, MA, United States). Sequestosome 1/p62 (5114) and LC3A/B (12741) antibodies were purchased from Cell Signaling Technology (Beverly, MA, United States). TFEB (AF7015) antibody was purchased from Affinity Biosciences (Melbourne, Australia), histone 3 (orb10805) antibody was acquired from Biorbyt (Cambridge, United Kingdom), and β-actin (60008-1-Ig) antibody was purchased from Proteintech (Wuhan, China). PrimeScript^TM^ RT reagent kit (Perfect Real Time) (RR037A) and TB Green^®^ Premix Ex Taq^TM^ II (Tli RNaseH Plus) (RR820A) were purchased from TaKaRa (Tokyo, Japan). Lipofectamine^TM^ 3000 transfection reagent (L3000008) was purchased from Thermo Fisher Scientific (Carlsbad, CA, United States) and the terminal deoxynucleotidyl transferase-mediated dUTP-biotin nick end labeling assay (TUNEL) assay kit (11684817910) was purchased from Roche (Penzberg, Germany).

### Cell Culture Conditions

Normal rat kidney epithelial-like (NRK-52E) cells, a cell line used widely in renal disease research, was purchased from the Type Culture Collection of the Chinese Academy of Sciences (Shanghai, China). The cells were maintained at 37°C in 5% CO2/95% air in Dulbecco’s Modified Eagle’s Medium (Hyclone, United States) supplemented with 10% fetal bovine serum (Gibco, Grand Island, NY, United States); after passaging, the cells were used for subsequent experiments. NRK-52E cells were pretreated with or without 32 μmol/L RSV for 2 h, and then treated with or without 800 μmol/L oxalate.

### Detection of Cell Viability and Apoptosis

The CCK-8 assay was used to evaluate the cell viability in accordance with the manufacturer’s protocol. Briefly, the NRK-52E cells were counted and seeded in 96-well plates at 2,000 cells per well. The cells were then treated with different concentrations of oxalate (0–1000 μM), RSV (0–128 μM), or transfected with TFEB small interfering RNA (siRNA) for 24 h; subsequently, the original culture medium was replaced with 100 μL medium containing 10% CCK-8 and cultured at 37°C for 3 h. Finally, the absorbance at 450 nm was measured using a microplate reader (Thermo Fisher Scientific, Massachusetts, United States).

The extent of cell apoptosis was determined using the Annexin V-FITC apoptosis detection kit. NRK-52E cells were seeded at a concentration of 1 × 105 cells/well in 6-well plates and incubated for 24 h. After NRK-52E cells were treated with oxalate or RSV or transfected with TFEB siRNA for 24 h, the cells were trypsinized, collected, and washed with phosphate-buffered saline (PBS, Hyclone). The washed cells were resuspended in binding buffer, incubated with 5 μL Annexin V-FITC and 5 μL propidium iodide in the dark for 10 and 5 min, respectively, and then analyzed immediately using a flow cytometer (BD FACSCalibur).

### ROS and MDA Assays

ROS levels in the intracellular and renal tissues of rats were detected using the ROS assay kit and DHE, respectively. NRK-52E cells were incubated in 96-well plates at 8,000 cells/well. After the NRK-52E cells were treated with oxalate or RSV or transfected with TFEB siRNA for 24 h, the cells were incubated with 10 μM DCFH-DA in the dark for 30 min, and washed three times with serum-free medium. The intracellular ROS levels were observed immediately and imaged under an optical microscope (IX51, Olympus, Tokyo, Japan). Frozen sections of rat kidney were incubated with 5 μL DHE in the dark for 30 min, washed with phosphate-buffered saline, sealed with anti-fluorescence quenching agent, and observed using a fluorescence microscope (ECLIPSE CI, Nikon, Tokyo, Japan). Finally, ImageJ software (NIH, MD, United States) was used to quantify the fluorescence intensity of each group.

In accordance with the manufacturer’s protocol, we used the lipid peroxidation MDA assay kit to detect the level of MDA, the product of lipid peroxidation. The MDA level of the samples was expressed in μmol/g. The final results were presented as the average of the three independent measurements.

### Western Blotting Analysis

NRK-52E cells or rats were subjected to different treatments. Nuclear protein, cytoplasmic protein, and total protein were extracted in RIPA lysis buffer supplemented with phenylmethanesulfonyl fluoride. Then, the concentration of protein in each sample was measured using the bicinchoninic acid assay. Subsequently, proteins were separated by 10% sodium dodecyl sulfate polyacrylamide gel electrophoresis and transferred to a polyvinylidene fluoride membrane. Non-specific binding to the membrane was blocked with 5% bovine serum albumin for 2 h and incubated overnight in one of the following primary antibodies: OPN (1:1000), BMP2 (1:800), IL-6 (0.8 μg/mL), LC3A/B (1:1000), Sequestosome 1/p62 (1:1000), TFEB (1:800), histone 3 (1:1000), and β-actin (1:500) at 4°C. The protein bands were washed three times with phosphate-buffered saline, incubated with horseradish peroxidase-coupled secondary antibodies for 1 h at room temperature, and visualized using an enhanced chemiluminescence assay kit. The band intensities were analyzed using ImageJ software (NIH, MD, United States).

### Cell Transfection and Real-Time Quantitative Polymerase Chain Reaction (RT-qPCR)

siRNA transfection was used to downregulate TFEB expression. The siRNA sequences used in this study were 5′-AGGCCGTCATGCATTACAT-3′ (TFEB siRNA) and 5′-TTCTCCGAACGTGTCACGTdTdT-3′ (NC siRNA), which were synthesized by RiboBio (Guangzhou, China). When the monolayer cell density in the 6-well plate reached 70–80% confluence, a plasmid DNA premix containing 2,500 ng was thoroughly mixed with Lipofectamine^TM^ 3,000 and incubated for 10–15 min before transfection into each well, as specified in the manufacturer’s instructions (Invitrogen).

Total cellular RNA was extracted with Trizol reagent (Thermo Fisher Scientific, Massachusetts, United States). Subsequently, mRNA was reverse transcribed into cDNA using the PrimeScript^TM^ RT reagent Kit (Perfect Real Time) and RT-qPCR was performed using the TB Green^®^ Premix Ex Taq^TM^ II (Tli RNaseH Plus) in an ABI Prism 7300 system (Thermo Fisher Scientific). The β-actin was used as the reference gene and 2^–ΔΔ*Ct*^ method was used to calculate the fold change of the target gene. All primer sequences used in the study are shown in Supplementary Table 1.

### Immunofluorescence Analysis

After NRK-52E cells were treated with or without RSV for 24 h, the cells were fixed with 4% paraformaldehyde for 15 min and permeabilized with 0.5% Triton X-100 PBS at room temperature for 20 min. The permeabilized cells were blocked with goat serum for 30 min at room temperature and incubated with TFEB antibody (1:100) at 4°C overnight. The cells were incubated with fluorescent secondary antibody at 20–37°C for 1 h and DAPI (Beyotime Institute of Biotechnology, Jiangsu, China) for 5 min. Finally, the cells were observed and imaged under a fluorescence microscope (BX53, Olympus, Tokyo, Japan).

### Transmission Electron Microscopy (TEM) Analysis

Rat renal tissue samples and NRK-52E cells were first fixed in 2.5% glutaraldehyde for 2–4 h and dehydrated with graded alcohol. The cells or ultrathin tissue sections were stained, observed, and imaged under an electron microscope (HT7800, HITACHI, Japan).

### Animal Experimental Design

Forty male Sprague-Dawley (SD) rats (6–8 weeks old, 200 ± 20 g) were purchased from Experimental Animal Research Center of Hubei (Wuhan, China) (SCXK: 2020-0018). These rats were randomly allocated into one of the following four groups: control (*n* = 10); glyoxylic acid monohydrate (GAM) treatment, which was the model group (*n* = 10); GAM + RSV treatment (*n* = 10); and GAM + MC treatment (*n* = 10). Rats in the control group received an intragastric administration of 5 mL/kg/day saline solution. In the GAM group, rats received an intraperitoneal injection of 100 mg/kg/day GAM and an intragastric administration of 5 mL/kg/day saline solution. In the GAM + RSV group, rats received an intraperitoneal injection of 100 mg/kg/day GAM and an intragastric administration of 10 mg/kg/day RSV (Oksay et al., 2017). In the GAM + MC group, rats received an intraperitoneal injection of 100 mg/kg/day GAM and an intragastric administration of 0.08% (w/v) MC. All rats were given free access to food and maintained in an environment of 25°C during the experimental period. After the experiment (8 days after the treatment), all animals were euthanized, and serum, 24-h urine, and kidney tissue specimens were collected from rats in each group. This study was approved by the Animal Care and Use Committee of Tongji Hospital, Tongji Medical College, Huazhong University of Science and Technology.

### Morphological and Biochemical Analyses

The weight of the rats was measured every 2 days. The oxalate assay kit was used in accordance with the manufacturer’s instructions to detect oxalate levels in the rat urine. Briefly, after the sample was prepared, the reaction mixture was added and incubated for 30 min, and the optical density at 450 nm was measured using a microplate reader (Thermo Fisher Scientific, Massachusetts, United States). The concentration of oxalate was then calculated from a standard curve. The calcium assay kit was used to detect the urinary calcium excretion levels and the procedures were similar to those described above. Finally, the optical density at 575 nm was measured on a microplate reader.

### Rat Kidney Histologic Analysis

The rat kidney tissue was fixed in 10% phosphate-buffered formalin solution and embedded in paraffin. The paraffin-embedded tissues were sliced into 5-μm sections for the subsequent experiments. In accordance with the manufacturer’s instructions, the TUNEL assay was used to detect apoptosis in rat kidney tissue, and the tissues were imaged under an optical microscope (Olympus, Tokyo, Japan). The pathological morphology of rat kidney tissue was observed by hematoxylin and eosin staining. In addition, we used immunohistochemical staining to detect the protein expression of OPN, BMP2, and IL-6 in the rat kidney tissue. Calcium crystal deposition in the rat kidney tissue was detected by von Kossa staining, and ImageJ software was used to quantify CaOx crystal deposition in renal tissue sections.

### Statistical Analysis

All results were performed independently at least three times. Statistical analysis for all data obtained was performed with SPSS 25.0 (SPSS Inc., Chicago, United States) statistical software. Tukey’s method with one-way ANOVA was used for experiments comparing multiple groups and a *t*-test was used for experiments comparing two groups. All data were shown as the mean ± standard deviations (SD), with a *P*-value of <0.05 considered statistically significant.

## Results

### Effects of RSV on Kidney Pathophysiology and Urine Biochemical Characteristics in GAM-Treated SD Rats

**FIGURE 1:**
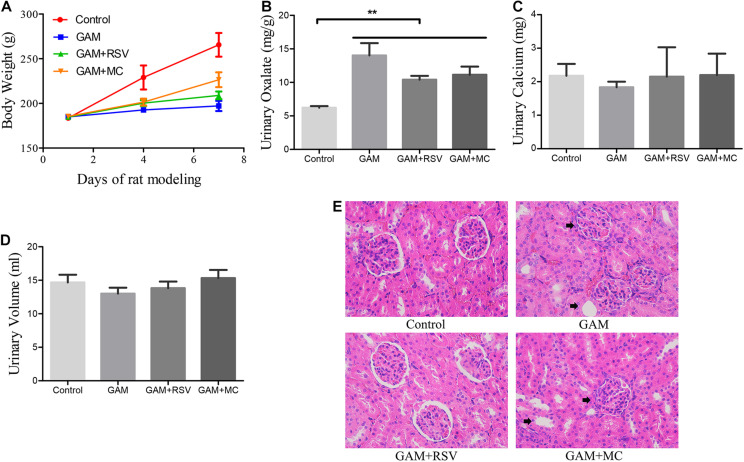
Effects of RSV on kidney pathophysiology and urine biochemical characteristics in GAM induced SD rats. **(A)** Body weight changes in each group after RSV treatment for 0–7 days. **(B)** Urinary oxalate excretion levels of rats in each group at the end of the experiment. **(C)** Urinary calcium excretion levels of rats in each group at the end of the experiment. **(D)** 24-h urine volumes of rats in each group at the end of the experiment. **(E)** HE staining showed pathological changes of renal tubules and glomeruli in each group at the end of the experiment (magnification ×400). Black arrows represent swollen and damaged renal tubule and glomerulus. **P < 0.01.

There were no significant differences in the body weight of rats in the GAM, GAM + RSV, and GAM + MC groups after treatment, but mean values were significantly lower than in the control group (Figure 1A). Urinary oxalate excretion was not significantly different among the GAM, GAM + RSV, and GAM + MC groups, but the mean values were significantly higher than in the control group (Figure 1B). However, there were no significant differences in urinary calcium excretion levels and 24-h urine volumes between all groups (Figures 1C,D). HE staining of rat kidney tissue revealed significant histological damage at the junction of the medullary and cortex in the GAM and GAM + MC groups, including glomerular edema, narrowing or even disappearance of renal capsule, dilatation, and necrosis of renal tubules. However, RSV treatment significantly reduced these renal injuries (Figure 1E).

### RSV Decreased Kidney CaOx Crystal Deposition and Osteogenic Protein Expression *in vivo*

**FIGURE 2:**
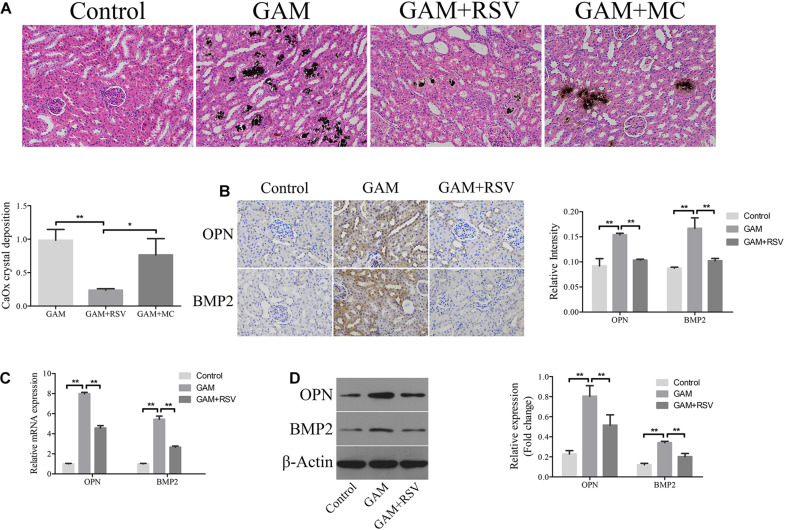
RSV decreased kidney CaOx crystal deposition and osteogenic related proteins expression in SD rat induced by GAM. **(A)** Von Kossa staining detected calcium crystal deposition at the junction of renal cortex and medulla in GAM-induced SD rat kidney (magnification 200×). Quantification of calcium crystal deposition level. **(B)** The expressions of OPN and BMP2 in rat kidney tissues were detected by immunohistochemical staining (magnification 400×). Quantification of immunohistochemical staining for OPN and BMP2 by Image Pro Plus software. **(C)** The expressions of OPN and BMP2 mRNAs were detected by RT-qPCR assay in rat kidney tissues. **(D)** The expressions of OPN and BMP2 proteins were detected by western blot assay in rat kidney tissues. Quantitative analysis of the densities of western blot bands. **P < 0.01.

von Kossa staining was used to detect CaOx crystal deposition levels in the renal tissues of rats in each group. Compared with the control group, greater CaOx crystal deposition occurred at the junction of the medullary and cortex of the rat kidney tissue in the GAM and GAM + MC groups, but there was no significant difference in CaOx crystal deposition between the GAM and GAM + MC groups (Figure 2A). However, after RSV treatment, CaOx crystal deposition was significantly reduced in the GAM + RSV group compared with the GAM and GAM + MC groups (Figure 2A). The immunohistochemical analysis results showed that the expression of osteogenic proteins OPN and BMP2 in the rat kidney tissue was higher in the GAM group than the control group (Figure 2B). RSV treatment, which plays a decisive role in protection, significantly reduced the expression of OPN and BMP2 (Figure 2B). The results of RT-qPCR and western blotting were similar to those above (Figures 2C,D).

### RSV Attenuated Overexpression Oxidant Species, Inflammation and Apoptosis, and Induced Activated Autophagy *in vivo*

**FIGURE 3:**
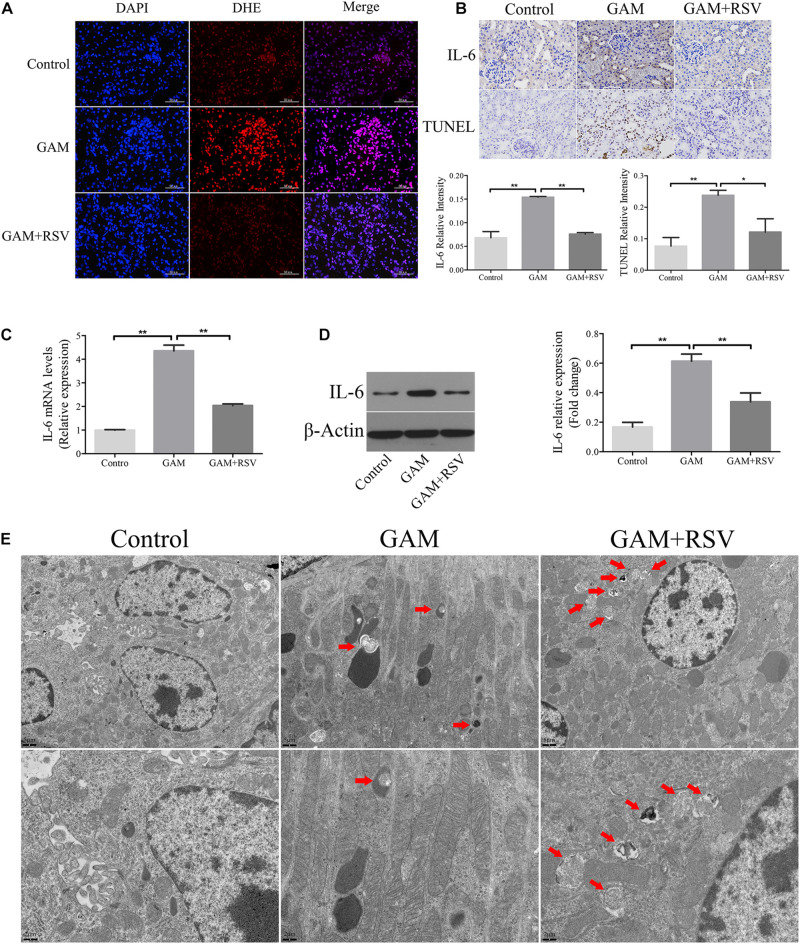
RSV attenuated oxidant species overexpression, inflammation and apoptosis, and induced autophagy activation in GAM-induced SD rat kidney. **(A)** DHE staining detected the overexpression of oxidant species in GAM-induced SD rat kidney. Scale bar: 50 μm. **(B)** The inflammatory cytokines IL-6 expression and apoptosis in GAM-induced SD rat kidney tissue were determined by immunohistochemical staining and TUNEL staining, respectively (magnification 400×). Quantification of immunohistochemical staining for IL-6 and TUNEL staining by Image Pro Plus software. **(C)** The expression of IL-6 mRNA was detected by RT-qPCR assay in rat kidney tissues. **(D)** The expression of IL-6 protein was detected by western blot assay in rat kidney tissues. Quantitative analysis of the densities of western blot bands. **(E)** Quantitative changes in autophagic vacuoles under TEM in kidney sections from GAM-induced SD rat. The red arrows indicate autophagic vacuoles. Scale bar: 5 and 2 μm. *P < 0.05, **P < 0.01.

DHE staining was used to detect the levels of oxidative stress in the renal tissues of rats in each group. The oxidative stress level in the kidney tissue in the GAM group was significantly higher than that in the control group (Figure 3A), and RSV treatment could significantly reduce the oxidative stress level in the GAM + RSV group (Figure 3A). Subsequently, the TUNEL assay and immunohistochemical staining were used to detect the extent of apoptosis and the expression of IL-6 in the renal tissues of rats in each group. The results showed that compared with the control group, the intraperitoneal injection of GAM significantly increased the level of renal apoptosis and the expression of IL-6, and RSV treatment significantly reduced the level of renal apoptosis and the expression of IL-6 induced by GAM (Figure 3B). The RT-qPCR and western blotting results were also similar (Figures 3C,D). Moreover, TEM analysis showed that the number of autophagic vacuoles (including autophagosomes and autolysosomes) was significantly higher in the GAM + RSV group than in the control and GAM groups (Figure 3E). These results indicated that RSV could reduce oxidative stress, inflammation, and apoptosis, and induce autophagy in the kidney tissue.

### RSV Reduced Oxidative Stress, Inflammation, and Osteogenic Protein Expression in Oxalate-Treated NRK-52E Cells

**FIGURE 4:**
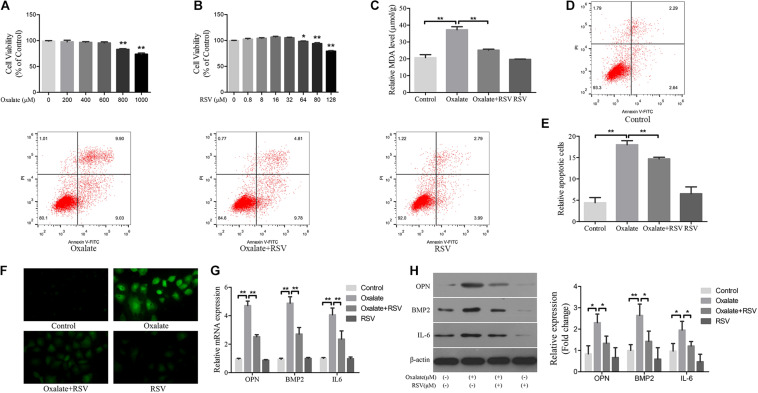
Effects of RSV on oxidative stress, inflammation, and osteogenic related proteins expression in NRK-52E cells induced by oxalate. **(A)** NRK-52E cells were treated with potassium oxalate at different concentrations (0–1000 μM) for 24 h. CCK-8 assay was used to detect NRK-52E cell activity. **(B)** NRK-52E cells were treated with RSV at different concentrations (0–128 μM) for 24 h. CCK-8 assay was used to detect NRK-52E cell activity. **(C)** Lipid peroxidation was evaluated by pretreatment of NRK-52E cells with or without RSV and detection of MDA level in the supernatant of cell lysate. **(D)** Flow cytometry was used to determine the proportion of apoptotic NRK-52E cells with or without RSV pretreatment. **(E)** The bar graphs showed the proportion of apoptotic cells in each group. **(F)** Fluorescence microscope was used to determine the production of ROS in cells NRK-52E with or without RSV pretreatment (magnification 400×). **(G)** The expressions of OPN, BMP2, and IL-6 mRNAs were detected by RT-qPCR assay in NRK-52E cells with or without RSV pretreatment. **(H)** The expressions of OPN, BMP2, and IL-6 proteins were detected by western blot assay in NRK-52E cells with or without RSV pretreatment. Quantitative analysis of the densities of western blot bands. *P < 0.05, **P < 0.01.

Hyperoxaluria has been identified as one of the most common risk factors for urinary stone disease (Coe et al., 2005). Oxidative stress induced by high oxalate concentrations has been reported to contribute to cell damage and promote CaOx crystal deposition in renal tissue (Aggarwal et al., 2013). We first investigated the effects of different concentrations of oxalate (0, 200, 400, 600, 800, and 1000 μM) on the viability of NRK-52E cells using the CCK-8 assay. A significant difference in viability occurred when the concentration reached 800 μM (Figure 4A), and this concentration was selected for subsequent experiments. Next, the CCK-8 assay was used to detect the effects of different RSV concentrations (0, 0.8, 8, 16, 32, 64, 80, and 128 μM) on the viability of NRK-52E cells and the results showed that RSV had no significant effect on cell viability at concentrations below 32 μM (Figure 4B). To further investigate the effects of RSV on oxalate-induced oxidative injury in NRK-52E cells, we measured MDA levels, which are markers of lipid peroxidation damage and oxidative stress. The results showed that, compared with the control group, the MDA level in NRK-52E cells treated with high concentrations of oxalate was significantly increased, whereas the MDA level in NRK-52E cells pretreated with RSV was significantly decreased (Figure 4C). The flow cytometry analysis showed that high concentrations of oxalate significantly increased the proportion of NRK-52E cells in early and late apoptosis, and RSV pretreatment significantly reduced the proportion of cells in early and late apoptosis (Figures 4D,E). In addition, the immunofluorescence assay showed that ROS levels were significantly increased in cells exposed to high concentrations of oxalate, and that RSV significantly inhibited intracellular ROS production (Figure 4F). Moreover, RT-qPCR results showed that the mRNA expression of BMP2, OPN, and IL-6 in NRK-52E cells treated with oxalate was significantly decreased after RSV treatment (Figure 4G). Western blotting analysis revealed similar results (Figure 4H). These results indicated that RSV could attenuate oxalate-induced oxidative stress and reduce inflammation and osteoblast-related protein expression.

### RSV Activated Autophagy in Oxalate-Treated NRK-52E Cells

**FIGURE 5:**
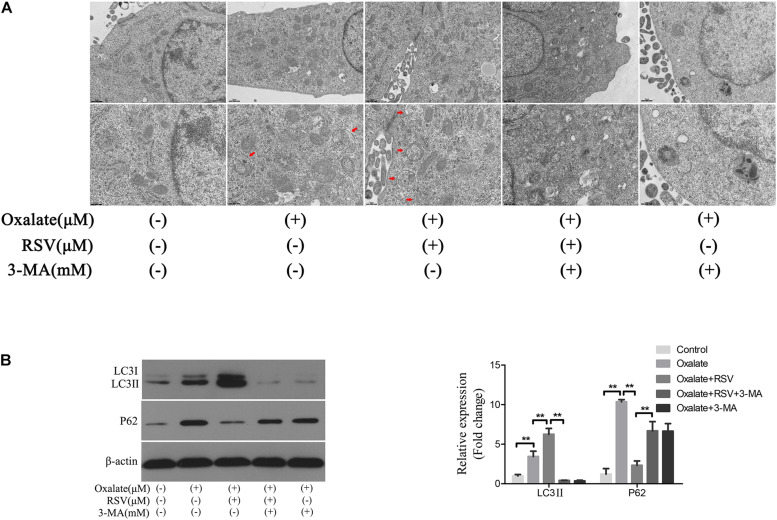
Effect of RSV on autophagy activity of NRK-52E cells exposed to oxalate. **(A)** The quantitative changes of NRK-52E autophagosome by TEM after the above treatment. The red arrows indicate autophagic vacuoles. Scale bar: 2 and 1 μm. **(B)** The expressions of LC3 II and P62 proteins were detected by western blot assay in NRK-52E cells with or without RSV or 3-MA pretreatment. Quantitative analysis of the densities of western blot bands. **P < 0.01.

To investigate the role of autophagy in the RSV-mediated attenuation of oxidative injury in oxalate-treated NRK-52E cells, the cells were exposed to oxalate following pretreatment with or without RSV for 2 h. TEM analysis showed that the number of autophagic vacuoles was significantly increased in the oxalate + RSV group compared with the control and oxalate groups (Figure 5A). The expression of the autophagy marker, LC3, and the selective autophagy key adaptor protein, p62, were detected by western blotting. The results showed that following oxalate treatment alone, expression of both p62 and LC3II was increased, suggesting that autophagic flux was inhibited. However, after RSV pretreatment, expression of LC3II was significantly increased and that of p62 was significantly decreased, suggesting that autophagic flux was activated (Figure 5B). In addition, to further confirm the RSV-induced activation of autophagy in NRK-52E cells treated with oxalate, the cells were pretreated with 3-MA, an inhibitor of early autophagy. The TEM analysis showed that 3-MA inhibited the formation of intracellular autophagic vacuoles induced by RSV (Figure 5A). Western blotting analysis showed that, compared with the oxalate + RSV group, the oxalate + RSV + 3-MA group exhibited decreased expression of LC3II and increased expression of p62, suggesting that autophagic flux was inhibited (Figure 5B). These results indicated that RSV could activate autophagy in NRK-52E cells exposed to oxalate.

### RSV Induced TFEB Activation in NRK-52E Cells

**FIGURE 6:**
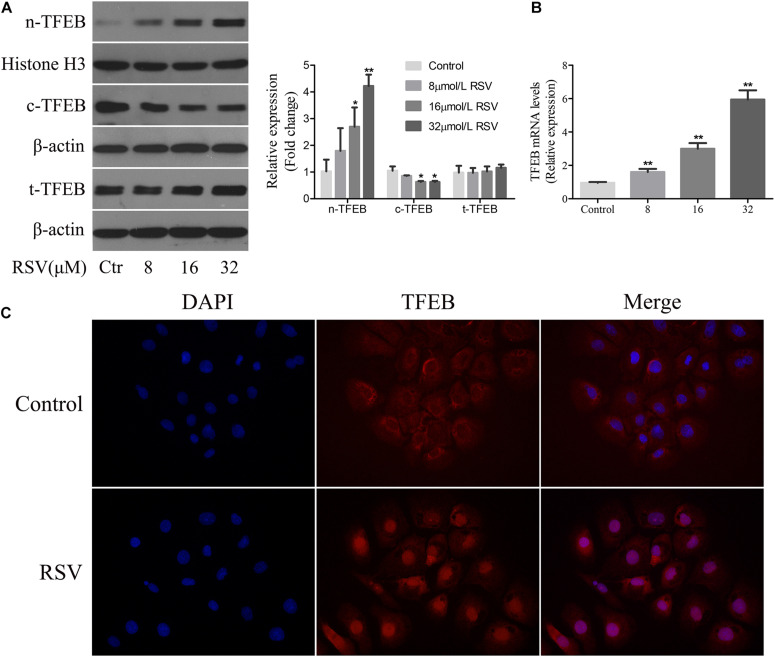
Effect of RSV on TFEB activity in NRK-52E cells. **(A)** NRK-52E cells were treated with RSV at different concentrations (0–32 μM) for 24 h. The expressions of nuclear TFEB (n-TFEB), cytoplasmic TFEB (c-TFEB), and total TFEB (t-TFEB) proteins were detected by western blot assay. Quantitative analysis of the densities of western blot bands. **(B)** NRK-52E cells were treated with RSV at different concentrations (0–32 μM) for 24 h. The expressions of n-TFEB, c-TFEB, and t-TFEB mRNAs were detected by RT-qPCR assay. **(C)** TFEB immunofluorescence assay results showed that the fluorescence intensity of TFEB antibody in nrK-52E cell nucleus increased after RSV treatment. *P < 0.05, **P < 0.01.

Transcription factor EB is a major regulator of the transcript of genes involved in autophagy and lysosomal biogenesis. Phosphorylated TFEB is an inactive form isolated in the cytoplasm; and when TFEB is dephosphorylated, it is translocated to the nucleus and regulates transcription of the target genes (Settembre et al., 2011, 2013). Following treatment of NRK-52E cells with different concentrations of RSV, western blotting analysis showed that there was no significant difference in the total expression of TFEB protein at different RSV concentrations, although TFEB expression increased gradually in the nucleus and decreased in the cytoplasm as the RSV concentration increased (Figure 6A). The RT-qPCR results showed that the mRNA expression of TFEB increased gradually (Figure 6B). Further immunofluorescence analysis showed that RSV increased intracellular nuclear migration of TFEB compared with the control group (Figure 6C). These results indicated that RSV could induce TFEB activation in NRK-52E cells.

### RSV Activated Autophagy in NRK-52E Cells Exposed to Oxalate in a TFEB-Dependent Manner

**FIGURE 7:**
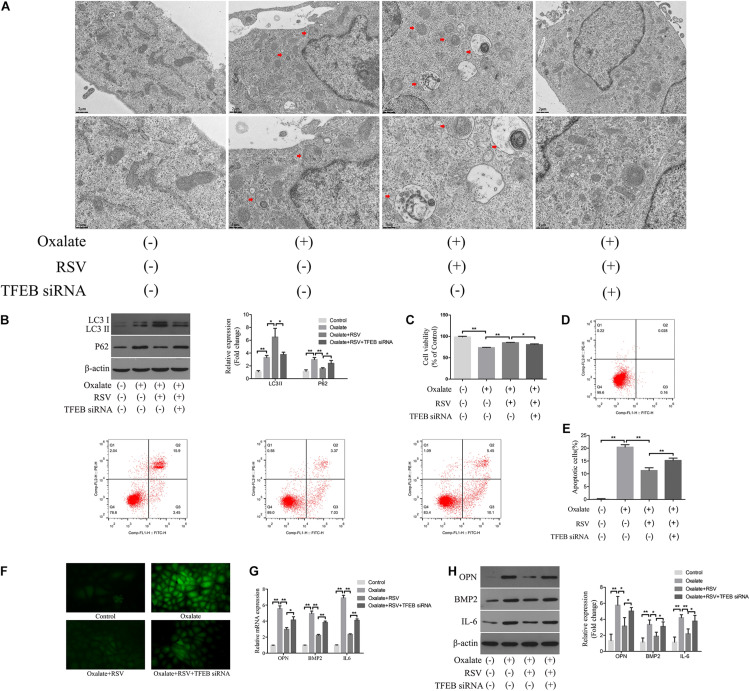
RSV activates autophagy of NRK-52E cells exposed to oxalate in a TFEB-dependent manner. **(A)** The quantitative changes of NRK-52E autophagosome by TEM with or without oxalate exposure, as well as RSV pretreatment or TFEB knockdown. The red arrows indicate autophagic vacuoles. Scale bar: 2 and 1 μm. **(B)** The expressions of LC3 II and P62 proteins were detected by western blot assay in NRK-52E cells with or without oxalate exposure, as well as RSV pretreatment or TFEB knockdown. Quantitative analysis of the densities of western blot bands. **(C)** CCK-8 assay was used to detect activity of NRK-52E cells with or without oxalate exposure, as well as RSV pretreatment or TFEB knockdown. **(D)** Flow cytometry was used to determine the proportion of apoptotic NRK-52E cells with or without oxalate exposure, as well as RSV pretreatment or TFEB knockdown. **(E)** The bar graphs showed the proportion of apoptotic cells in each group. **(F)** Fluorescence microscope was used to determine the production of ROS in cells NRK-52E with or without oxalate exposure, as well as RSV pretreatment or TFEB knockdown (magnification 400×). **(G)** The expressions of OPN, BMP2, and IL-6 mRNAs were detected by RT-qPCR assay in NRK-52E cells with or without oxalate exposure, as well as RSV pretreatment or TFEB knockdown. **(H)** The expressions of OPN, BMP2, and IL-6 proteins were detected by western blot assay in NRK-52E cells with or without oxalate exposure, as well as RSV pretreatment or TFEB knockdown. Quantitative analysis of the densities of western blot bands. *P < 0.05, **P < 0.01.

To further investigate the regulatory role of TFEB in the RSV-induced autophagy response, we transfected TFEB siRNA into NRK-52E cells and conducted subsequent experiments (Supplementary Figure 2). TEM analysis showed that RSV pretreatment significantly increased the number of autophagic vacuoles in NRK-52E cells exposed to oxalate, whereas transfection of TFEB siRNA significantly reduced the number of autophagic vacuoles in NRK-52E cells compared with RSV pretreatment alone (Figure 7A). The western blotting analysis also showed that the RSV-induced upregulation of LC3II and downregulation of p62 were significantly inhibited by TFEB siRNA transfection in NRK-52E cells exposed to oxalate, suggesting that autophagic flux was inhibited (Figure 7B). Next, we explored the effects of transfection of TFEB siRNA in NRK-52E cells on RSV protection. The results showed that the RSV-induced increase in the viability of NRK-52E cells (Figure 7C) and decrease in cells apoptosis level (Figures 7D,E) were significantly inhibited by transfection of TFEB siRNA in NRK-52E cells exposed to oxalate. In addition, the immunofluorescence analysis showed that the RSV-induced decrease in intracellular ROS production was significantly reversed after transfection with TFEB siRNA in NRK-52E cells exposed to oxalate (Figure 7F). The RT-qPCR results showed that transfection of TFEB siRNA in NRK-52E cells exposed to oxalate significantly attenuated the RSV-mediated inhibition of IL-6, OPN, and BMP2 mRNA expression (Figure 7G). The western blotting analysis revealed similar results (Figure 7H). Collectively, these results indicated that RSV reduced oxidative stress, inflammation, and osteogenic protein expression in oxalate-treated NRK-52E cells through the activation of a TFEB-dependent autophagy pathway.

## Discussion

In the present study, we found that RSV significantly decreased CaOx crystal deposition in the rat kidney through the inhibition of oxidative stress, the inflammatory response, and the expression of the osteogenic proteins BMP2 and OPN in renal tubular epithelial cells. Further analysis revealed the involvement of autophagy in this process: RSV induced protective autophagy, at least in part, through activation of the TFEB signaling pathway, thereby attenuating oxidative stress and inflammation in renal tubular epithelial cells. These results revealed the regulatory role of autophagy in oxalate-induced oxidative injury and in CaOx crystal deposition in renal tubular epithelial cells and the kidney tissue, and suggested a new mechanism through which RSV attenuated oxidative injury and CaOx crystal deposition.

It is established that oxidative stress-induced injury is involved in the pathophysiological mechanisms of a variety of chronic diseases, including neurodegenerative diseases (Chen and Zhong, 2014), cardiovascular diseases (Kattoor et al., 2017), and urolithiasis (Zhu et al., 2019). Moreover, various phytochemicals or drugs with antioxidant activities have been reported to exert protective or therapeutic effects against these diseases in cell and animal studies (Bulucu et al., 2008; Kim et al., 2011; Jin et al., 2014; Zhu et al., 2019). Several studies have confirmed the anti-oxidative stress effect of RSV in different diseases. Cosín-Tomàs et al. (2019) found that RSV promoted the expression of antioxidant genes and anti-aging factors in lymphoblastoid cell lines, which induced resilience and protection against Alzheimer’s disease. RSV has various effects in vascular systems, it is reported to inhibit the proliferation of vascular smooth muscle cells, promote autophagy, improve vascular function in the elderly, and moderately reduce systolic blood pressure in patients with hypertension and blood sugar in patients with diabetes (Pollack et al., 2017; Breuss et al., 2019). In our study, we treated NRK-52E cells with 800 μM oxalate to simulate the hyperoxaluria microenvironment, in conjunction with pretreatment with or without RSV. Our results showed that the intracellular ROS level, extent of apoptosis, and release of MDA were significantly reduced by RSV pretreatment. *In vivo* animal experiments revealed similar results in the kidney tissue from rats administered RSV. Moreover, several studies have reported that osteogenic proteins, such as BMP2 and OPN, may have an important role in nephrolithiasis (Jia et al., 2014; Khan et al., 2014). These *in vivo* and *in vitro* results from our current study revealed that RSV could reduce the expression of the osteogenic proteins OPN and BMP2, as well as that of the inflammatory factor IL-6, thereby attenuating the calcium crystal deposition in renal tissue. These results suggested that RSV does exert antioxidant activity and protective effects against kidney stone formation. However, the specific mechanism through which RSV exerts these effects was not clarified, and further study is required.

Autophagy is an intracellular self-degradation process that removes misfolded proteins, damaged organelles such as the mitochondria, endoplasmic reticulum, peroxisomes, and pathogens through lysosomal-mediated degradation (Glick et al., 2010). Autophagy plays an important role in a variety of diseases, including cancer (Onorati et al., 2018), neurodegenerative diseases (Scrivo et al., 2018), and cardiovascular diseases (Mialet-Perez and Vindis, 2017), and has been reported to be involved in the cytoprotective effects of phytochemicals (Delmas et al., 2011). Various studies have reported that RSV exerts its anti-inflammatory, antioxidant, and tumor-suppressive effects by inducing protective autophagy (Kou and Chen, 2017; Pourhanifeh et al., 2019; Tian et al., 2019). Therefore, we believe that autophagy is essential for the multiple biological protective effects of RSV. In this study, we investigated the role of autophagy in the RSV-induced protection of renal tubular epithelial cells and preventive effects against kidney stone formation. In a rat model of CaOx kidney stone formation, we first found that the autophagic vacuoles in renal tissues increased significantly after RSV supplementation. Then, the *in vitro* cell experiments showed that RSV pretreatment significantly upregulated the expression of LC3II and downregulated the expression of p62 in NRK-52E cells exposed to oxalate, suggesting that autophagic flux was activated. However, 3-MA significantly reversed the RSV-induced upregulation of LC3II expression and downregulation of p62 expression in oxalate-treated NRK-52E cells. These results suggested that autophagy activation was advantageous and crucial for the RSV-mediated attenuation of oxalate-induced oxidative damage and calcium crystal deposition in NRK-52E cells. These results were similar to previous studies in which autophagy activation protected renal tubular epithelial cells from cisplatin, cyclosporin A, or urinary protein damage (Liu et al., 2014). However, this was in contrast to the results of exposure to ischemia/reperfusion injury, tunicamycin, or oxalate (Havasi and Dong, 2016; Duan et al., 2018). Autophagy has a dual role in many diseases: in cases of mild injury, autophagy inhibits cell apoptosis and damage by degrading damaged or senescent organelles, re-establishes homeostasis, and plays a role in cell survival; in contrast, in cases of severe damage, excessive activation of autophagy may be deleterious and responsible for non-apoptotic cell death (Nishida et al., 2008; Luo and Rubinsztein, 2010; Li et al., 2017). Conversely, the effect of autophagy on cells is related to the duration of the process. It has been found that autophagy induced by unilateral ureteral obstruction has a renoprotective effect in the early stages of kidney obstruction (Kim et al., 2012), whereas cytokines, growth factors, and oxidative stress can induce the death of proximal tubular epithelial cells through excessive autophagy and apoptosis under the stimulation of persistent unilateral ureteral obstruction (Li et al., 2010). Therefore, the difference in these results may be related to the degree of cell damage caused by stress factors and the duration of autophagy; as such, the mechanism of autophagy in oxidatively damaged renal tubular cells requires further study. These results suggested that the activation of autophagy is crucial to enable RSV to exert its antioxidant activity and preventive effects against kidney stone formation. However, the exact mechanism of RSV-induced autophagy activation remains unclear.

Transcription factor EB is an important transcription factor that comprehensively regulates the expression of lysosomal and autophagosomal biogenic genes (Napolitano and Ballabio, 2016). Chao et al. (2018) found that increased TFEB expression promoted the biogenesis of lysosomes and autophagy, and protected mice from ethanol-induced liver damage and steatosis. In this study, we explored whether RSV induced the activation of autophagy by the regulation of TFEB activity. After treatment with increasing concentrations of RSV, the ratio of nuclear to cytoplasmic TFEB was found to increase gradually, and the immunofluorescence results also suggested that RSV promoted intracellular TFEB nuclear migration. TEM and western blotting analysis showed that the RSV-induced upregulation of LC3II and downregulation of p62 was significantly inhibited by the transfection of TFEB siRNA in oxalate-treated NRK-52E cells, indicating that autophagic flux was inhibited. Concomitantly, intracellular ROS production and apoptosis were increased, and expression of IL-6 and the osteogenic proteins OPN and BMP2 was increased, suggesting that the anti-inflammatory action and anti-oxidative stress effects of RSV were weakened after TFEB expression was silenced. These results suggest that RSV exerts its anti-oxidative stress activity and preventive effects against kidney stone formation by activating TFEB-induced autophagy. However, the specific mechanism of TFEB-regulated autophagy in renal tubular epithelial cells requires further exploration.

Our study revealed a new mechanism by which RSV exerts its anti-oxidative stress activity and preventive effects against kidney stone formation in renal tubular cells, and identified the important role of autophagy in this mechanism. However, there were some limitations to our study: it is necessary to explore whether there are other mechanisms by which RSV induces renal tubule cell autophagy, and the specific mechanism through which TFEB regulates autophagy in renal tubular cells should be determined.

In conclusion, through *in vivo* and *in vitro* experiments, our study further confirmed the protective effect of RSV against the pathological process of kidney stone formation. In addition, we have presented the first evidence that RSV exerts its anti-oxidative stress activity and preventive effects against kidney stone formation, at least in part, through the activation of TFEB-induced autophagy (Graphical Abstract). Therefore, RSV may be an effective drug for the prevention of renal tubular injury and to prevent kidney stone formation.

## Data Availability Statement

The raw data supporting the conclusions of this article will be made available by the authors, without undue reservation.

## Ethics Statement

The animal study was reviewed and approved by the Animal Care and Use Committee of Tongji Hospital, Tongji Medical College, Huazhong University of Science and Technology.

## Author Contributions

YW conceived, designed, and conducted the research. YX, JZ, HH, and BQ conducted the research and revised the manuscript. TW and SW revised the manuscript. CL and YL designed the research, revised the manuscript, and had primary responsibility for final content. All authors read and approved the final manuscript.

## Conflict of Interest

The authors declare that the research was conducted in the absence of any commercial or financial relationships that could be construed as a potential conflict of interest.

## Abbreviations

CaOx: calcium oxalate
RSV: resveratrol
TFEB: transcription factor EB
MTOR: mechanistic target of rapamycin kinase
ROS: reactive oxygen species
3-MA: 3-methyladenine
DHE: dihydroethidium
GAM: glyoxylic acid monohydrate
MC: methyl cellulose
MDA: malondialdehyde
NRK-52E: normal rat kidney epithelial-like cell
SD: sprague-Dawley
DMEM: dulbecco’s modified eagle medium
PBS: phosphate buffered saline
FBS: fetal bovine serum
BSA: bovine serum albumin
CCK-8: cell counting kit-8
TEM: transmission electron microscope
SDS-PAGE: sodium dodecyl sulfate-polyacrylamide gel electrophoresis
PVDF: polyvinylidene difluoride
siRNA: small interfering RNA
TUNEL: terminal deoxynucleotidyl transferase-mediated dUTP-biotin nick end-labeling assay
OPN: osteopontin
IL-6: interleukin 6
BMP2: bone morphogenetic protein 2.
