# Supplementary material for: Resveratrol Attenuates Oxalate-Induced Renal Oxidative Injury and Calcium Oxalate Crystal Deposition by Regulating TFEB-Induced Autophagy Pathway
Source: Front Cell Dev Biol. 2021 Feb 25;9:638759. doi: 10.3389/fcell.2021.638759 (PMC7947311; doi:10.3389/fcell.2021.638759)
Supplement: Supplementary file 1 [file Data_Sheet_1.docx]

**Resveratrol attenuates oxalate-induced renal oxidative injury and calcium oxalate crystal deposition by regulating TFEB-induced autophagy pathway**

| Name | Primer | Sequence |
| --- | --- | --- |
| Rat β-actin | Forward | 5‘- ACCATCGGGAATGAACGCTT -3’ |
|  | Reverse | 5‘- CTGTCAGCAATGCCTGGGTA -3’ |
| Rat OPN | Forward | 5‘- CCAGCCAAGGACCAACTACA -3’ |
|  | Reverse | 5‘- GCTGGCAGTGAAGGACTCAT -3’ |
| Rat BMP2 | Forward | 5‘- CATGGGTTTGTGGTGGA -3’ |
|  | Reverse | 5‘- TTTGTGTTTGGCTTGACG -3’ |
| Rat IL-6 | Forward | 5‘- CTGCTCTGGTCTTCTGGAGTT -3’ |
|  | Reverse | 5‘- AAGTGCTGCTACCCTGAGATG -3’ |
| Rat TFEB | Forward | 5‘- TGGGCAAATCCCTTCTGT -3’ |
|  | Reverse | 5‘- GTTGATTCCGGCTCCCT -3’ |

Supplementary Tab. S1. The sequences of primer RNA


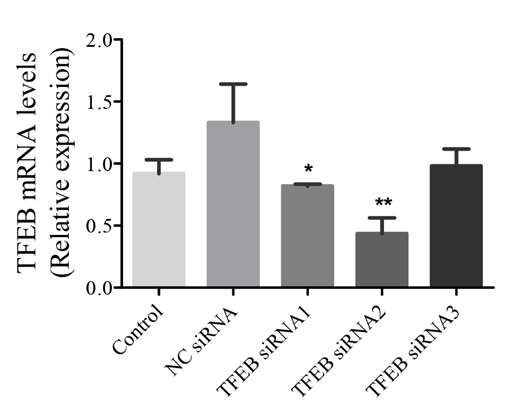


Supplementary Fig. S1. Transcript levels for TFEB were assessed following transfection of scrambled control RNA (NC siRNA) or TFEB-specific siRNA (TFEB siRNA1-3) in NRK-52E. **P* < 0.05 versus NC siRNA group, ***P* < 0.01 versus NC siRNA group.
